# Biological behaviour of human umbilical artery smooth muscle cell grown on nickel-free and nickel-containing stainless steel for stent implantation

**DOI:** 10.1038/srep18762

**Published:** 2016-01-04

**Authors:** Liming Li, Liwen An, Xiaohang Zhou, Shuang Pan, Xin Meng, Yibin Ren, Ke Yang, Yifu Guan

**Affiliations:** 1 Institute of Biotechnology, Northeastern University, Shenyang, China; 2 Department of Biochemistry and Molecular Biology, China Medical University, Shenyang, China; 3 Institute of Metal Research, Chinese Academy of Sciences, Shenyang, China

## Abstract

To evaluate the clinical potential of high nitrogen nickel-free austenitic stainless steel (HNNF SS), we have compared the cellular and molecular responses of human umbilical artery smooth muscle cells (HUASMCs) to HNNF SS and 316L SS (nickel-containing austenitic 316L stainless steel). CCK-8 analysis and flow cytometric analysis were used to assess the cellular responses (proliferation, apoptosis, and cell cycle), and quantitative real-time PCR (qRT-PCR) was used to analyze the gene expression profiles of HUASMCs exposed to HNNF SS and 316L SS, respectively. CCK-8 analysis demonstrated that HUASMCs cultured on HNNF SS proliferated more slowly than those on 316L SS. Flow cytometric analysis revealed that HNNF SS could activate more cellular apoptosis. The qRT-PCR results showed that the genes regulating cell apoptosis and autophagy were up-regulated on HNNF SS. Thus, HNNF SS could reduce the HUASMC proliferation in comparison to 316L SS. The findings furnish valuable information for developing new biomedical materials for stent implantation.

Vascular stent implantation has become a routine surgical procedure for treatment of coronary artery diseases^1^. In spite of its success in saving a great number of patients, vascular stent implantation demonstrates several limitations. Statistical analysis has indicated that, within one year after primary stent implantation, more than 20% of stent-implantation patients will develop in-stent restenosis (ISR) unless anticoagulation therapy will be taken routinely. This ISR has been a serious complication to stent surgical practice^2^,^3^. At present, the most commonly used metallic materials for intravascular stents are the medical grade 316L stainless steel (316L SS) and cobalt-based alloys such as L605 and MP35N^4^. They have demonstrated excellent mechanical properties and biocompatibilities. However, the high nickel content (usually 10–14%) in these stent materials has been suspected to be the primary cause for the acute thrombosis and long-term restenosis because the released nickel and chromium ions in body environment have allergic and toxic effects^5^,^6^,^7^,^8^, which might trigger the ISR process^9^,^10^. These negative outcomes have raised concerns from the cardiovascular surgeons as well as vascular stent makers^9^,^10^,^11^,^12^.

Scientists and engineers in material science have devoted a great effort to develop new types of stent materials with a hope of eliminating the allergic and inflammatory effects caused by nickel ions. Drug eluting stents (DES) have been developed in the late 1990s^13^. These pharmacological agents embedded in the polymer layer are primarily focused on suppressing vascular smooth muscle cell (SMC) proliferation^14^. However, DES shows the late stent thrombosis due to delayed endothelialization. On the other hand, new types of stainless stent materials such as high nitrogen nickel-free austenitic stainless steel (HNNF SS) have been invented^4^,^15^,^16^,^17^. It has shown attractive mechanical properties, better corrosion resistance and good biocompatibility^15^,^16^,^17^,^18^,^19^. In previous study, we have evaluated the biological effects of this nickel-free stainless steel material. We compared the cellular behaviour (proliferation, cell cycle and apoptosis) of human umbilical vein endothelial cell (HUVEC) cultured on HNNF SS and 316L SS. We also examined the expression profiles of several genes regulating cell proliferation and apoptosis, and proposed biological mechanism underlying these cellular behaviour^20^.

The abnormal proliferation of SMCs underneath the endothelial monolayer is closely related with many types of artery diseases, including the ISR process. Unfortunately, the detailed mechanisms underlying the ISR process are yet to be determined^21^,^22^. Exposure of SMC to the nickel ions released from the stent materials is suspected to delay the stent endothelialization and lead to the subsequent development of ISR^23^,^24^. Several studies have examined the biological properties of SMCs in the ISR-related biological processes previously. However, these studies utilized either polymer-coated DESs^25^,^26^,^27^ or the SMC of animal models^28^. Thus, experimental results and derived conclusions could not be applied directly to the human applications. The biological responses of human SMCs to nickel-free HNNF SS have never been thoroughly investigated.

The objective of this study is to examine the biological responses of primary human umbilical artery smooth muscle cell (HUASMC) to HNNF SS and 316L SS at molecular level and cellular level. After seeding HUASMCs on HNNF SS and 316L SS, we evaluated the cellular behaviour of proliferation, apoptosis and cell cycle. Then, we examined the expression profiles of several genes participating in the cell cycle and cell apoptosis, and proposed that the apoptotic and autophagic events might delay the HUASMC cell proliferation on HNNF SS. Our studies enrich our understanding about the biological behaviour of human SMC and human endothelial cell in in-stent restenosis, and provide an experimental basis for future development of novel biomedical materials for stent applications.

## Results

### Cell adhesion

**Figure 1:**
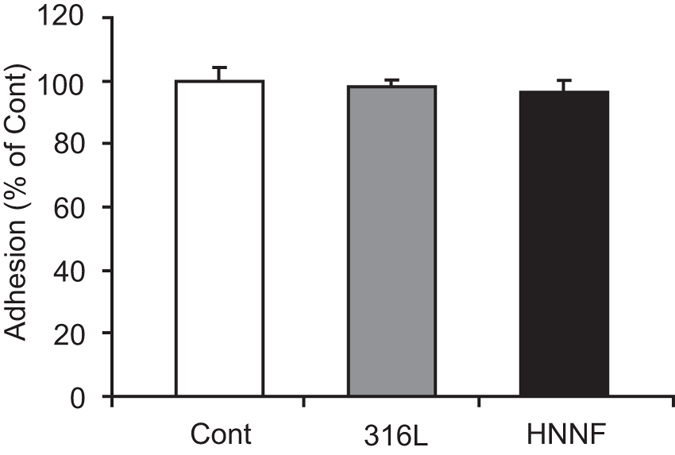
Adhesion of HUASMCs to 316L SS substrate, HNNF SS substrate and Type-IV collagen-coated culture dish (referred to as control). Data are presented as mean ± SD.

HUASMCs were seeded on HNNF SS and 316L SS surfaces. Four hours later, cells were harvested and stained with trypan blue, and the cell number was counted under microscope. As shown in Fig. 1, the percentages of cells adhered on surfaces of HNNF SS and 316L SS are almost identical to that of the control (HUASMCs cultured in Type-IV collagen coated culture dish).

### Cell proliferation

**Figure 2:**
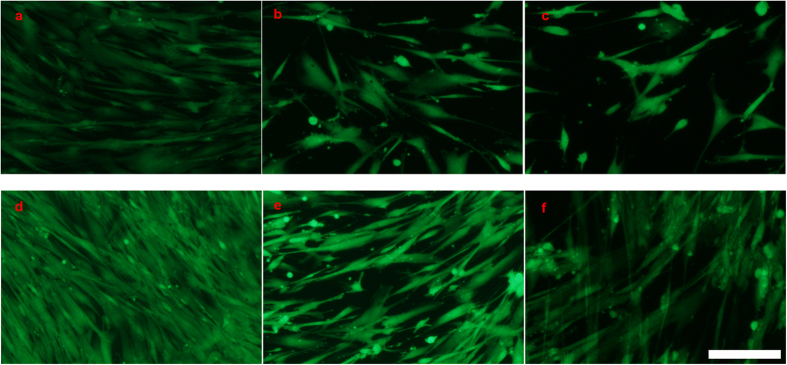
Fluorescent images of HUASMCs grown on different surfaces of materials. Culture for 3 days in Type-IV collagen-coated culture dish (referred to as control) (**a**), 316L SS (**b**) and HNNF SS (**c**), and for 7 days in Type-IV collagen-coated culture dish (referred to as control) (**d**), 316L SS (**e**) and HNNF SS (**f**). Scale Bars = 100 μm.

HUASMCs cultured on the steel surfaces were stained with Calcein-AM and their images were recorded under a fluorescence microscope after 3-day and 7-day periods (Fig. 2). The images showed that HUASMCs grew uniformly on the steel surfaces as a monolayer, no obvious difference in cell morphology was observed in comparison with the control. Calcein-AM is the dye which can stain only the living cells. The number of cells grown on HNNF SS and 316L SS was increased with the culture time. After 3-day culture, the HUASMC cellular spreading was good on the steel surfaces, and after 7-day culture, HUASMCs covered evenly the entire steel surfaces. Figure 2 shows that the number of the living HUASMCs on different surfaces follows the order of HNNF SS <316L SS <control.

**Figure 3:**
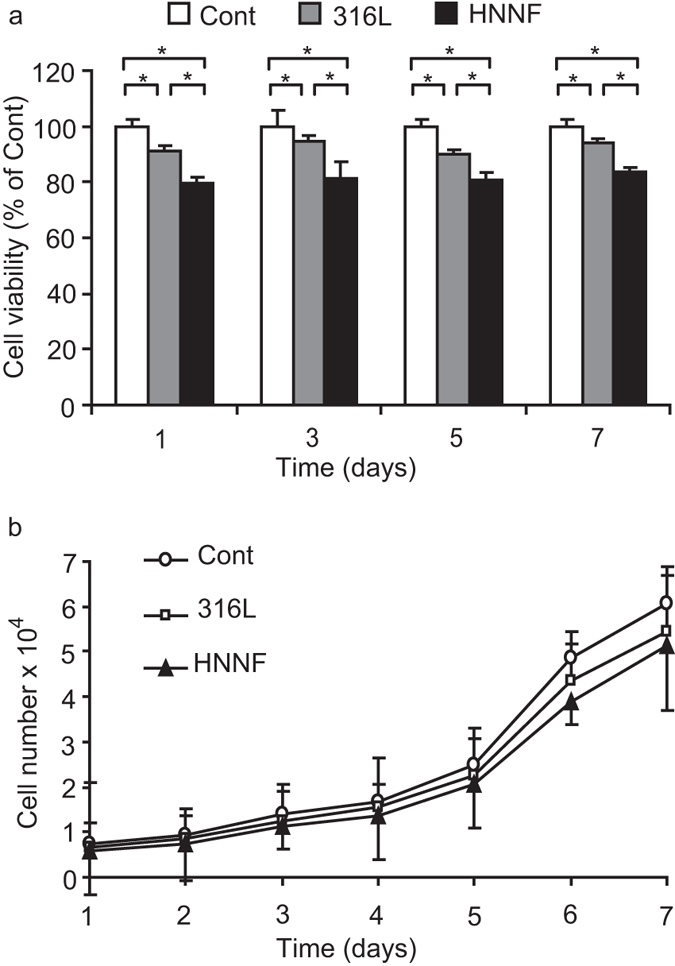
Measurement of HUASMC proliferation. (**a**) Relative growth rates of HUASMCs on 316L SS and HNNF SS after 1-, 3-, 5- and 7-day growth with respect to the control of HUASMCs (100%). (**b**) Growth curve of HUASMCs on surfaces of 316L SS and HNNF SS, and in Type-IV collagen-coated culture dish (referred to as control) in a 7-day period. Data are presented as mean ± SD. *Indicate statistically significant difference at p < 0.05.

The proliferation ability of HUASMCs on different steel surfaces was also compared. HUASMCs were seeded on each steel surface for different period and then assayed using CCK-8 method. As shown in Fig. 3a, cell viability follows this trend: HNNF SS <316L SS <control.

The cell proliferation behaviour was also tested in terms of the absolute cell numbers. During a 7-day period, the absolute number of HUASMCs grown on different steel surfaces was counted and the growth curves were plotted. As illustrated in Fig. 3b, it became evident that HUASMCs cultured on HNNF SS were less than those on 316L SS after 3 days, and the latter was further less than those of the control.

### Cell cycle of synchronized HUASMCs

**Figure 4:**
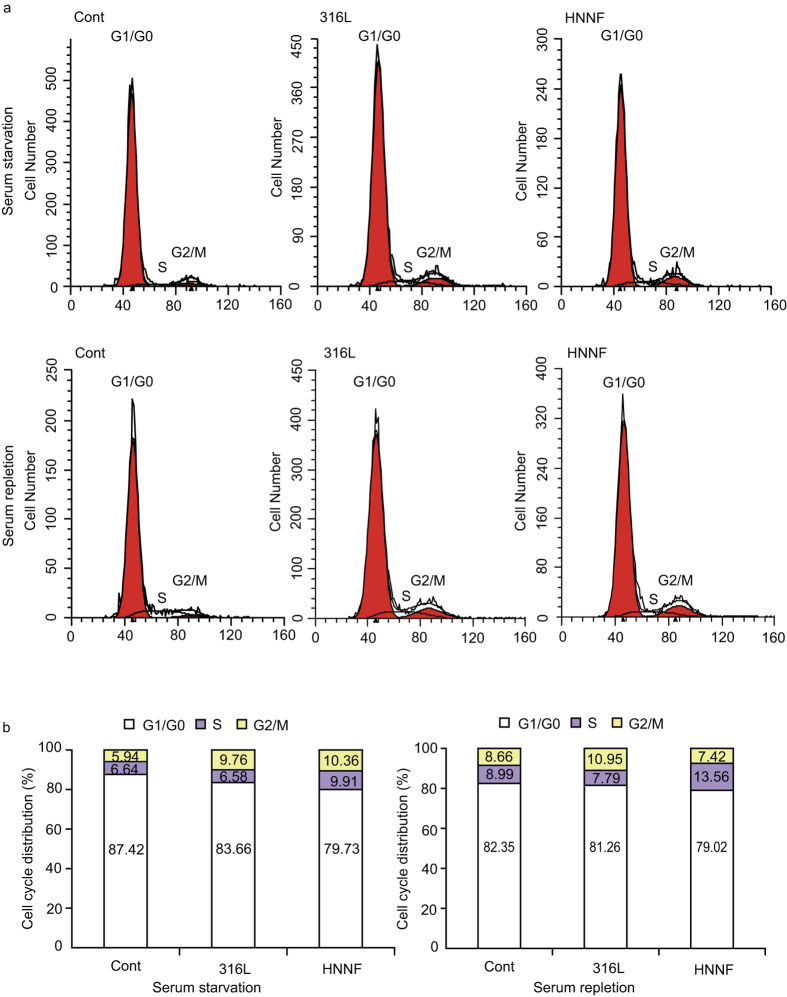
Effect of 316L SS and HNNF SS on cell cycle progression of HUASMCs. (**a**) Flow cytometric data showing the cell cycle distributions of HUASMCs cultured on 316L SS and HNNF SS, respectively, without FBS for 22 h (top panel), and cell cycle distributions of HUASMCs cultured with the whole culture medium for 24 h after cell synchronization (bottom panel). (**b**) Histographic representations of the cell cycle distributions of HUASMCs.

The influence of HNNF SS and 316L SS on HUASMC cell cycle progression was analyzed (Fig. 4). The serum-deprivation approach was used to synchronize HUASMC cultured on HNNF SS and 316L SS. The serum-deprivation for 22 h has led to 79.73 ± 3.63%, 83.66 ± 2.47% and 87.42 ± 4.66% of HUASMC to be synchronized in G1/G0 phase for HNNF SS, 316L SS and the control, respectively. After serum-retreatment for 24 h, the cell populations in the G1/G0 phase were reduced to 79.02 ± 2.76%, 81.26 ± 0.64% and 82.35 ± 1.89% for HNNF SS, 316L SS and the control, respectively.

The HUASMC populations in other phases of the cell cycle were also different on these two different stent materials (Fig. 4b). Before and after serum retreatment, HUASMC populations in S phase were increased from 9.91% to 13.56% (by 36.8%) for HNNF SS, and from 6.58% to 7.79% (by 18.4%) for 316L SS. In G2/M phase, the HUASMC population on HNNF SS decreased from 10.36% to 7.42% (by 28.4%) before and after serum treatment, whereas the same number changed from 9.76% to 10.95% (by 12.2%) on 316L SS.

**Figure 5:**
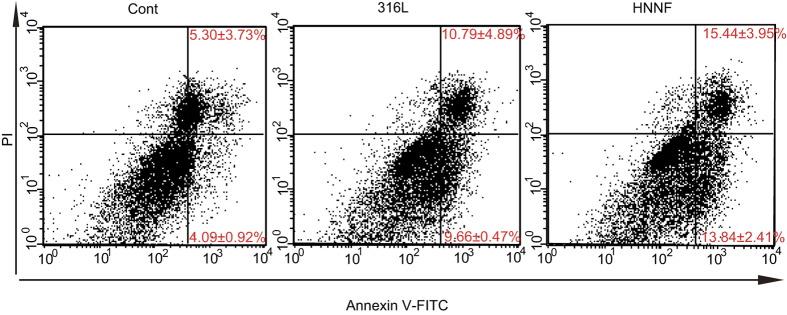
Flow cytometric analysis of apoptosis of HUASMCs. Cells were cultured in Type-IV collagen-coated culture dish (referred to as control), on surfaces of 316L SS and HNNF SS for 7 days. Data are presented as mean ± SD.

Flow cytometric analysis was performed to examine the apoptotic behaviour of HUASMC (Fig. 5). The ratios of the early apoptotic HUASMC were calculated to be 13.84 ± 2.41% and 9.66 ± 0.47% for HNNF SS and 316L SS, respectively, both higher than that of the control (4.09 ± 0.92%) (*p* < 0.05). Also, the ratios of the late apoptotic HUASMC were calculated to be 15.44 ± 3.95% and 10.79 ± 4.89% for HNNF SS and 316L SS, respectively, both slightly higher than that of the control (5.30 ± 3.73%) (*p* > 0.05).

### Quantitative real-time PCR analysis of gene expression of HUASMCs

**Figure 6:**
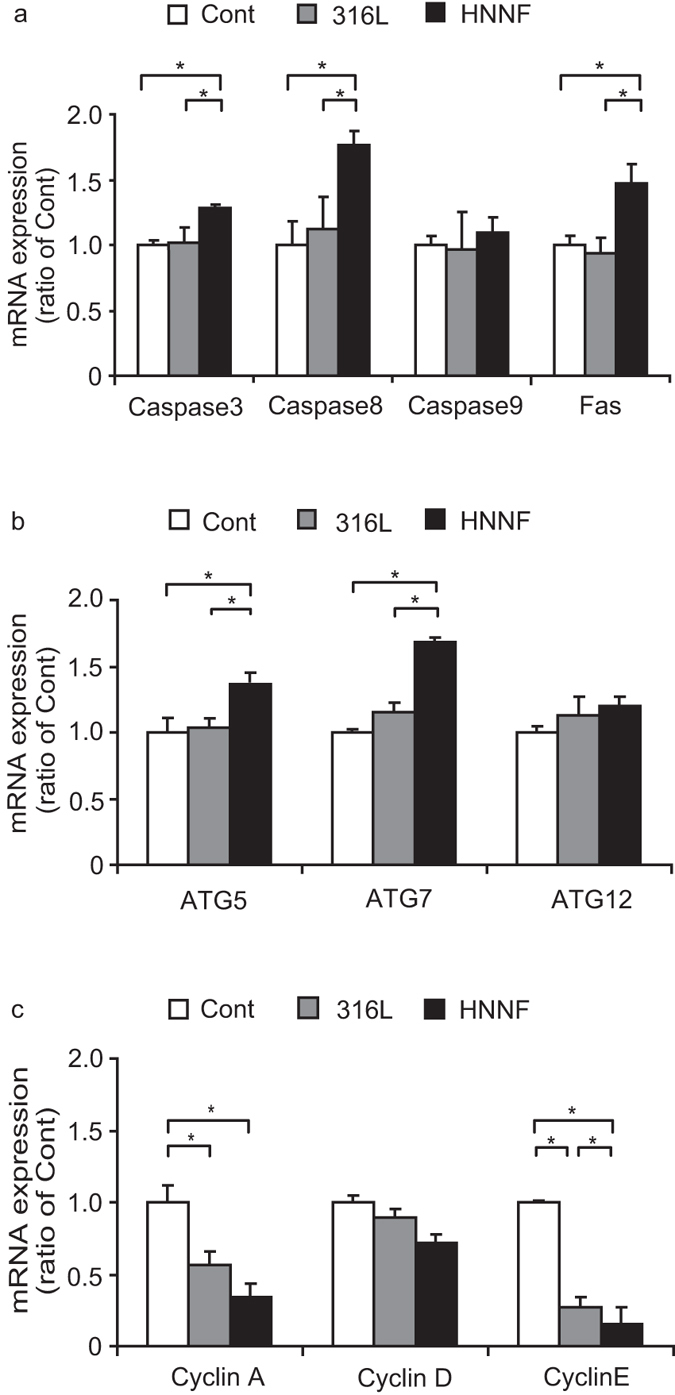
Gene expression profiles of HUASMCs. Cells grew in Type-IV collagen-coated culture dish (referred to as control), on surfaces of 316L SS and HNNF SS for 7 days determined using qRT-PCR method. Data are presented as mean ± SD. *indicate statistically significant difference at p < 0.05.

As illustrated in Fig. 6, when HUASMCs were cultured on HNNF SS, genes caspase-3, caspase-8 and Fas exhibited an up-regulated expression in comparison with the control (*p* < 0.05). On the other hand, when HUASMCs were cultured on 316L SS, caspase-3, caspase-8, caspase-9 and Fas were changed slightly within the experimental errors (*p* > 0.05).

ATG5 and ATG7 of HUASMC on HNNF SS also demonstrated an up-regulated expression by ~40% and ~70% with respect to the control (*p* < 0.05). However, when cultured on 316L SS, the expression levels of these genes were slightly changed within the experimental errors (*p* > 0.05).

Genes participating in the cell cycle also demonstrated different expression profiles. In comparison with the control, cyclin E and cyclin A of HUASMC on HNNF SS were down-regulated by 80% and 65% (*p* < 0.05). In the case of 316L SS, these two genes were down-regulated by 70% and 45% (*p* < 0.05). In contrast, cyclin D of HUASMC on HNNF SS and 316L SS was both slightly down-regulated (*p* > 0.05).

## Discussion

Vascular stent implantation has been widely used for treatment of arterial trauma. However, bare metallic stent as an external stimulus could provoke allergic and inflammatory responses. It has been speculated that the abnormal proliferation of SMCs underneath the endothelial cell monolayer is the primary cause of the ISR formation. In general, the mechanical stress of the inner wall of the artery vessel during the stent implantation induces the inflammation and the corresponding neointima formation^14^. The SMC proliferation could be continued even after the heal of implantation, which triggers a series of biological events of platelet deposition, thrombin activation, thrombus aggregation, and restenosis eventually^22^. To avoid the ISR formation, new types of stent materials are expected to be able to stimulate the endothelial cell growth for rapid endothelialization and prevent the SMC proliferation. HNNF SS is one of the newly invented nickel-free stainless steels demonstrating a great potential for stent implantation. Our previous study has compared the biological responses of HUVEC to HNNF SS and 316L SS, respectively, and found that HNNF SS is indeed able to advance the HUVEC cell endothelialization on the stainless steel surface^20^. In the present study, we make a further study on the HUASMCs. SMC proliferation on different stent materials has been explored previously. For instance, a water-soluble non-anticoagulant dextran (E9) has been found to be able to inhibit the SMC proliferation via activating the MAPK pathway^29^. However, these results are not highly satisfactory, since these experiments have utilized SMC of rats^28^,^29^,^30^,^31^ or rabbits^29^,^32^,^33^, or stent materials coated with polymer^25^,^26^,^27^, growth factor^28^ and even inhibitor^34^. A previous study has examined the expression patterns of the surface markers of human and rabbit mesenchymal stem cells, and found significant differences^35^. Moreover, some drug therapies have been tested for prevention of ISR. These drugs demonstrated beneficial effects when tested on animal models of restenosis, but have been proven unsuccessful in clinical trials^36^. These data highlight the shortcomings of conventional animal cells and the necessity of using human SMC for such studies.

In this study, we observed that HUASMC proliferated more slowly on HNNF SS than on 316L SS (Fig. 3), suggesting a beneficial effect of HNNF SS in reducing SMC proliferation and possibly preventing the ISR occurrence. In the present study, we also found that the cell cycle had been influenced by different stent materials. The flow cytometric data (Fig. 4) indicated that more HUASMC on HNNF SS were attenuated in S phase, in contrast, more HUASMC on 316L SS progressed into the G2/M phase for cell division, meaning that HUASMC on HNNF SS takes a longer time to prepare enough materials for cell division than those on 316L SS. These flow cytometric results showed small changes of HUASMC populations in each phase before and after cell synchronization, but they are reasonable when considering the fact that the ISR development is a long term outcome. Similar data have been reported in other studies^37^,^38^. Overall, these results are consistent with the observed cell growth curves.

As indicated, the ISR development is a slow and long-term process of the stent implantation^13^. It is believed that the changes of gene expression occur far prior to any observable changes at the cellular level^37^. Thus, the gene expression profiles of HUASMC on different stent materials might be a good indicator for cellular behaviour, and the gene expression analysis were performed using qRT-PCR approach. Our qRT-PCR showed that cyclin E and cyclin A of HUASMCs on HNNF SS were down-regulated in comparison with those on 316L SS (*p* < 0.05) (Fig. 6c). It has been known, during the cell cycle, cyclin D and cyclin E initiate DNA replication, and cyclin A stimulates DNA replication. Thus, the low level of these cyclin proteins showed a direct and consistent correlation with the delayed cell cycle progression as well as the reduced proliferation of HUASMC on HNNF SS. Realizing the significance of cell cycle for preventing vascular SMC proliferation and restenosis after arterial injury, Segev *et al.* have conducted comparative experiments to investigate the effect of p15^Ink4^, a member of the INK4 family of CDK inhibitors, on cell proliferation, cell cycle progression, and intimal hyperplasia. Their experimental results showed that over-expression of p15^Ink4^ inhibited cell cycle progression and subsequent in-stent intimal hyperplasia^32^. The different gene expressions on the nickel-free and nickel-containing stent materials in this study could be due to the influence of nickel ions.

There are two apoptotic pathways for cells to balance the growth and death. The first one is the death-receptor pathway, also known as extrinsic pathway. It is activated through a binding of specific protein ligands (also known as death ligands, such as FasL and TNF-α) to a cell surface transmembrane receptors such as endogenous death receptors^39^. This binding leads to the recruitment of Fas-associated death domain (FADD), and then activates caspase-8 and subsequent downstream effectors^40^. The second apoptotic pathway is the mitochondrial pathway. It is usually initiated by stimuli such as cytotoxic drugs, heat shock, ionizing radiation, hypoxia, viral infections and other cellular stresses. All of the aforementioned stimuli provoke the release of mitochondrial components (such as cytochrome c) to prime a caspase-9-activating complex in the cytosol^41^. Both pathways converge on a common downstream pathway, which mediates the final morphological and biochemical alterations of apoptosis that are characteristic of gene caspase-3^40^.

In the current study, we used flow cytometry to examine the apoptotic behaviour of cells. As shown in Fig. 5, more HUASMCs underwent death via the apoptotic pathway when seeded on HNNF SS than on 316L SS in early apoptosis (*p* < 0.05). The higher apoptotic rate might correlate with the lower proliferation rate of HUASMCs on HNNF SS. To interpret the observed apoptotic behaviour of HUASMC, qRT-PCR analysis was performed. The qRT-PCR results showed that genes caspase-8 and FAS were overexpressed on HNNF SS, whereas they were just at the basal level on 316L SS (Fig. 6a). These data indicate that the death-receptor pathway of HUASMC was activated more on HNNF SS than on 316L SS. Gene caspase-9 was expressed almost equally on HNNF SS and 316L SS surfaces, suggesting that the mitochondrial pathway was at the basal level. Consequently, as a downstream gene of the death-receptor pathway and the mitochondrial pathway, caspase-3 of HUASMC was expressed differently on HNNF SS and 316L SS surfaces (Fig. 6a). These data suggest that the HNNF SS may serve as the exogenous factor to activate the death-receptor pathway to induce apoptosis.

Autophagy is a newly discovered process providing a cell survival advantage under nutrient deprivation or other stress conditions. Autophagy could have a crosstalk with apoptotic pathway to exert a collective effect on cell proliferation^42^,^43^. Protein Atg5 involved in autophagy can also induce the caspase activation by interacting with the adaptor protein FADD, a component of the extrinsic apoptotic pathway^42^,^44^,^45^,^46^.

The qRT-PCR results showed that two important autophagic regulator genes, ATG5 and ATG7 of HUASMC, were up-regulated on HNNF SS, but they were expressed at the basal level on 316L SS (Fig. 6b). It seems that Atg7 promotes the apoptotic activation, since Atg7 facilitates the dimerization of Atg5 and Atg12 protein, and then affects the downstream effectors. Our data in this study showed that the HUASMC proliferation might be the collective consequence of the apoptotic nature and the autophagy.

Possible intracellular signal pathways of vascular SMC for neointima formation have also been explored in other studies^23^,^29^,^32^. Vascular endothelial growth factor (VEGF) appears to have a protective function by inhibiting the VSMC proliferation directly. This effect could occur via the mitogen-activated protein kinase pathway^47^,^48^,^49^,^50^. Experiment from Kim *et al.* suggested that lysophosphatidic acid (LPA) receptors couple to G protein Gq regulated proliferation through possible activation of ERK1/2 and Akt in VSMCs^51^. Activation of vascular SMC p38α MAPK is known to be required for neointimal development perhaps because of its ability to promote Rb phosphorylation and MCM6 expression^23^. Both ras and the non-ras pathways are also reported to contribute to the neointimal hyperplasia and restenosis^33^.

In this study, we investigated the biological behaviour of HUASMCs cultured on nickel-free HNNF SS and nickel-containing 316L SS, respectively, at cellular and molecular levels. Experimental data showed that HNNF SS could reduce the HUASMC proliferation in comparison to 316L SS. This observation is consistent with the cell cycle behaviour and the cell apoptotic property indicated by flow cytometric results. Quantitative RT-PCR analyzed the gene expression profiles of HUASMCs cultured on HNNF SS and 316L SS, and the genes participating in the cell cycle and cell apoptosis demonstrated a consistent trend with that of observed cellular behaviour. From the results of this study and our previous study^20^, HNNF SS seems to enhance the HUVEC proliferation for rapid endothialiazation and to retard the HUAMSC growth (although not able to promote the HUAMSC quiescence), suggesting the beneficial advantage of HNNF SS for ISR prevention. It should also be kept in mind that endothelial cell and SMC differentiate differently even exposed to an identical environment, and it is not surprising that these two type of cells behaviour different due to their intrinsic cellular characteristics.

However, experimental results and conclusions obtained in this study are still very preliminary. First, HNNF SS and 316L SS are different not only in the nickel content but also in the composition of other elements, thus, the biological effects of other major elements such as N, Mo, Si, … should not be simply ruled out. Therefore, the overall responses of endothelial cells and SMCs to different metal ions need a thorough analysis. Secondly, changes in cellular behaviour and consequent ISR development are a consequence of a long-term and constant influence by metal ions, and can only be notable in a long term observation. Thus, the *in vitro* study in a short period could not provide a comprehensive knowledge about the ISR mechanisms. Thirdly, the in-stent restenosis is an extreme complex biological event, and the stent material is just one effector. The influence of other biological factors (such as inflammatory factors, allergic effect, cell migration, …) and other biophysical factors (such as the shear stress of blood stream on endothelial cells, blood stream turbulence, …) have not been considered yet in most studies, and the collective impact of these factors on the in-stent restenosis has not been reported. Furthermore, the cellular behaviour must be accompanied by certain signal transduction pathways, and metal ions and other elements might be the activators or the inhibitors for particular steps along these pathways. However, such issues have not well characterized so far. Therefore, systematic studies on the cell regulation pathways and long-term effects of such factors on the in-stent restenosis should be conducted. The data and conclusions in this study provide a preliminary but very useful knowledge to guide the further investigations and development of novel biomaterials for stent implantation applications.

## Materials and Methods

### Materials

The HNNF SS used in this study, 00Cr18Mn15Mo2N (0.5-0.9) developed by Institute of Metal Research, Chinese Academy of Sciences, was melted in a 50 kg pressuring induction melting furnace under the protection of pure nitrogen gas, and its chemical composition was analyzed as C: 0.026; N: 0.62; Cr: 18.62; Mn: 15.8; Mo: 2.78; Si: 0.18; S: 0.004; P: 0.013 wt% and Fe in balance. The 316L SS for comparison, with analyzed composition of C: 0.025; Cr: 17.5; Mn: 1.06; Mo: 2.66; Ni: 13.07; Si: 0.6; S: 0.008; P: 0.02 wt% and Fe in balance, was melted in a 25 kg vacuum induction melting furnace according to ASTM F138-2003. These two steels were forged and rolled to billets for further processing, and a solution treatment was conducted before experiment to obtain a homogeneous austenitic microstructure. All the samples were cut from the rolled materials and were solution treated at 1373 K for 1 h, followed by water quenching, which then were machined into Ф32×1 mm or Ф12×1 mm slices, respectively, to fit to the 6-well or 24-well culture plates. All the slice samples were grounded with serial SiC papers, electrochemically polished and finally ultrasonically cleaned for 15 min in acetone for three times to remove any impurities on the surface. Prior to experiments, all the samples were ultrasonically cleaned with alcohol, rinsed in distilled water and then sterilized at 121 °C for 20 min.

### Cell culture

Primary human vascular SMCs from umbilical cord arteries were isolated by standard protocols^52^. HUASMCs were maintained in DMEM medium (Hyclone, Beijing, China) supplemented with 10% fetal bovine serum (FBS) (Gibco, Grand Island, USA), 100 IU/ml penicillin and 100 μg/ml streptomycin, in a humidified incubator with 5% CO_2_ at 37 °C.

### Cell adhesion

HUASMCs were seeded on surfaces of HNNF SS and 316LSS steel at a concentration of 5×10^4^ cells/cm^2^ (24-well culture plate). After 4-hour incubation, the non-adhered cells were discarded with the medium, and the steel surfaces were washed twice with phosphate-buffered saline (PBS) gently. Cells adhered on the surfaces were harvested with 0.25% trypsin and the number of cells was counted under an optical microscope with trypan blue. HUASMCs cultured in Type-IV collagen-coated dish were used as control throughout the whole study unless specified. Results were expressed as a percentage of the number with respect to that of the control.

### Observation of cell morphology and density

HUASMCs were seeded on the steel surfaces at a density of 5,000 cell/cm^2^ (24-well culture plate). Cells were cultured in DMEM medium containing 10% FBS in a humidified incubator (5% CO_2_ atmosphere at 37 °C) for 3 days and 7 days without renewing culture medium^38^,^53^. After each culture period, 0.5 μM Calcein-AM (Dojindo Laboratories, Kumamoto, Japan) was added to the medium, and cells were incubated at 37 °C for another 30 min. Excess Calcein-AM was removed by several washes with PBS. Images of cells were taken under a fluorescence microscope (Olympus IX-71, Olympus Optical Co., Tokyo, Japan).

### Measurement of cell proliferation

To assess the cell viability, HUASMCs were seeded on different steel surfaces at a concentration of 5,000 cells/cm^2^ (24-well culture plate), and cultured in an incubator (5% CO_2_ and at 37 °C) for 1-, 3-, 5- and 7-day periods. The cell viability was examined using the Cell Counting Kit-8 (CCK-8; Dojindo, Kumamoto, Japan) assay which was based on the principle of CCK-8 (water-soluble tetrazolium salt) cleavage to formazan-class dye by mitochondrial succinate tetrazolium reductase in viable cells. The cell counting solution of 50 μl was added into each well and mixed for 4 h, and then formazan-class dyes were detected by measuring absorbance at 450 nm using a TECAN Infinite M200 microplate reader (Tecan Group Ltd., Maennedorf, Switzerland). Cell viability was expressed as the ratio of the signal obtained from the cells incubated on steel surfaces^54^.

To assess the cell proliferation directly, the number of cells was counted. HUASMCs were seeded on different steel surfaces at a concentration of 5,000 cell/cm^2^ (24-well culture plate). During a 7-day culture incubation, the cells grown on each surface were harvested at each day and washed with PBS. The number of cells was then counted under an optical microscope with trypan blue.

### Cell cycle analysis and apoptosis assessment by flow cytometry

HUASMCs were seeded on different steel surfaces at a concentration of 6×10^4 ^cells/cm^2^ (6-well culture plate). After cells were cultured in an incubation with DMEM medium containing 10% FBS for 24 h, DMEM + 10% FBS was replaced with DMEM medium (without FBS). After serum starvation for 22 h, cells were treated with DMEM + 10% FBS for 24 h. At the points of serum starvation for 22 h and serum-retreated for 24 h, cells on the steel surfaces were trypsinized, washed with PBS and fixed in ice-cold 70% ethanol overnight. Cells were then treated with RNase at 37 °C and stained with propidium iodide (PI) solution (KeyGEN Biotech, Nanjing, China) for 30 min at 4 °C. PI-stained nuclei were analyzed with a flow cytometry (FACSCalibur, BD Biosciences, USA). The ratios of the cells in G1/G0, S and G2/M phases were calculated.

For cells apoptotic analysis, HUASMCs were seeded on different steel surfaces at a concentration of 5,000 cells/cm^2^ (6-well culture plate) and cultured in an incubator with DMEM medium + 10% FBS for 7 days. Cells were harvested, washed twice with ice-cold PBS and resuspended in the dark with AnnexinV-FITC and PI (KeyGEN Biotech, Nanjing, China) buffer for 15 min at room temperature. Cells were then analyzed with a flow cytometry. Cells were considered to be apoptotic when they were either Annexin V+/PI- (early apoptotic) or Annexin V+/PI+ (late apoptotic). All flow cytometry data were analyzed using the Mod Fit LT software (Verity Software House, Topsham, MN).

### Quantitative real-time PCR

HUASMCs were seeded on different steel surfaces at a concentration of 5,000 cells/cm^2^ (6-well culture plate) and cultured in an incubator with DMEM medium +10% FBS for 7 days. Total RNA of HUASMCs was isolated using TRIzol reagent (Invitrogen, Carlsbad, USA), reverse transcription (RT) was performed with the RT reagent kit (Takara Biotechnology Co., Ltd. Dalian, China), and quantitative real-time PCR (qRT-PCR) was performed using the SYBR Premix Ex Taq II (Takara Biotechnology Co., Ltd. Dalian, China). All these preparations were performed following the manufacturer’s protocols. The qRT-PCR oligonucleotide primers used in this study are shown in Table 1. The amplification and analysis were performed on a Real-Time PCR system (ABI prism 7500, Applied Biosystems, Foster City, USA). The PCR cycle was as follows: 95 °C for 30 sec, 40 cycles of 95 °C for 5 sec and 60 °C for 34 sec and followed by one cycle at 95 °C for 15 sec, at 60 °C for 1 h and at 95 °C for 15 sec. The qRT-PCR data were analyzed with a ΔΔC_t_ method and normalized using GAPDH cDNA as an internal control.

### Statistical analysis

Data analysis was performed using SPSS software (Chicago, Illinois, USA). All the experimental data were expressed as means ± standard deviation (SD) (n = 3), and statistically analyzed. The statistical significance of the results was performed by one-way analysis of variance (ANOVA) followed by Tukey’s post hoc multiple comparison tests. A *p* value less than 0.05 was considered statistically significant.

## Additional Information

**How to cite this article**: Li, L. *et al.* Biological behaviour of human umbilical artery smooth muscle cell grown on nickel-free and nickel-containing stainless steel for stent implantation. *Sci. Rep.* **6**, 18762; doi: 10.1038/srep18762 (2016).

## Figures and Tables

**Table 1:** Nucleotide sequences of primers used for qRT-PCR.

Gene	Primer notation	Sequence
GAPDH	Forward	GCACCGTCAAGGCTGAGAAC
	Reverse	TGGTGAAGACGCCAGTGGA
Caspase3	Forward	GACTCTGGAATATCCCTGGACAACA
	Reverse	AGGTTTGCTGCATCGACATCTG
Caspase8	Forward	CATTTGCATATTTAGCCGCCAAG
	Reverse	TTAAGAGTCCCAGGAATTCAGCAAC
Caspase9	Forward	GCCATATCTAGTTTGCCCACACC
	Reverse	CACTGCTCAAAGATGTCGTCCA
Fas	Forward	CAACAACCATGCTGGGCATC
	Reverse	TGATGTCAGTCACTTGGGCATTAAC
Cyclin A1	Forward	GAAATTGTGCCTTGCCTGAGTG
	Reverse	TCTGATATGGAGGTGAAGTTCTGGA
Cyclin D	Forward	ATGTTCGTGGCCTCTAAGATGA
	Reverse	CAGGTTCCACTTGAGCTTGTTC
Cyclin E	Forward	GCCGTTTACAAGCTAAGCAGCAG
	Reverse	CCAGATAATACAGGTGGCCAACAA
Atg5	Forward	TTGAATATGAAGGCACACCACTGAA
	Reverse	GCATCCTTAGATGGACAGTGCAGA
Atg7	Forward	CTGTAACTTAGCCCAGTACCCTGGA
	Reverse	TACGGTCACGGAAGCAAACAAC
Atg12	Forward	AGTAGAGCGAACACGAACCATCC
	Reverse	CCACGCCTGAGACTTGCAGTA
